# Apneias Muito Longas em Posição Prona em uma Paciente Eutrófica com Doença Arterial Coronariana: Implicações para o Risco Cardiovascular

**DOI:** 10.36660/abc.20200191

**Published:** 2021-02-02

**Authors:** Sofia Fontanello Furlan, Viktor Sinkunas, Pedro Rodrigues Genta, Geraldo Lorenzi, Luciano F. Drager

**Affiliations:** 1 Universidade de São Paulo Instituto do Coração São PauloSP Brasil Universidade de São Paulo Instituto do Coração, São Paulo, SP – Brasil

**Keywords:** Angioplastia, Apneia do Sono Tipo Obstrutiva, Doença da Artéria Coronariana, Intervenção Coronária Percutânea, Índice de Massa Corpórea, Obesidade, Monitoração Ambulatorial da Pressão Arterial, Oximetria

## Introdução

A apneia obstrutiva do sono (AOS) é uma condição clínica comum, caracterizada por obstruções recorrentes das vias aéreas superiores durante o sono, promovendo hipóxia intermitente e fragmentação do sono.^1^ Fatores de risco tradicionais incluem sexo masculino e obesidade. De maneira geral, pacientes com AOS grave apresentam eventos respiratórios mais longos e hipoxemia mais acentuada. A posição supina está consistentemente associada a índices mais graves de AOS em adultos.^2^ No entanto, a relação entre índices de AOS e posição prona é inconsistente.^2^ Aqui, relatamos uma apresentação muito peculiar da AOS caracterizada por eventos respiratórios muito longos em posição prona em uma paciente do sexo feminino, eutrófica, com histórico de hipertensão arterial, diabetes mellitus, doença renal crônica sob terapia dialítica e diagnóstico recente de doença arterial coronariana (DAC).

## Relato de Caso

**Figura 1:**
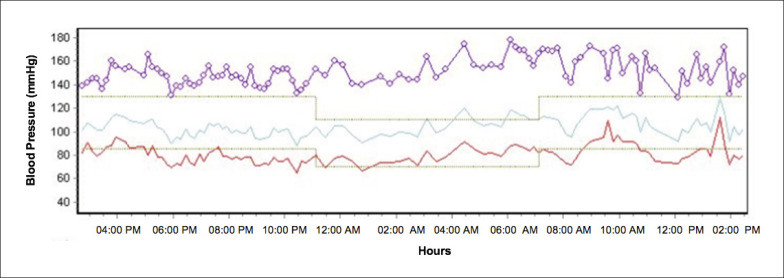
Monitoração ambulatorial da pressão arterial mostrando padrão de descenso reverso da pressão arterial sistólica. Pressão arterial média durante o dia: 150x81 mmHg; Pressão arterial média noturna: 155x79 mmHg.

**Figura 2:**
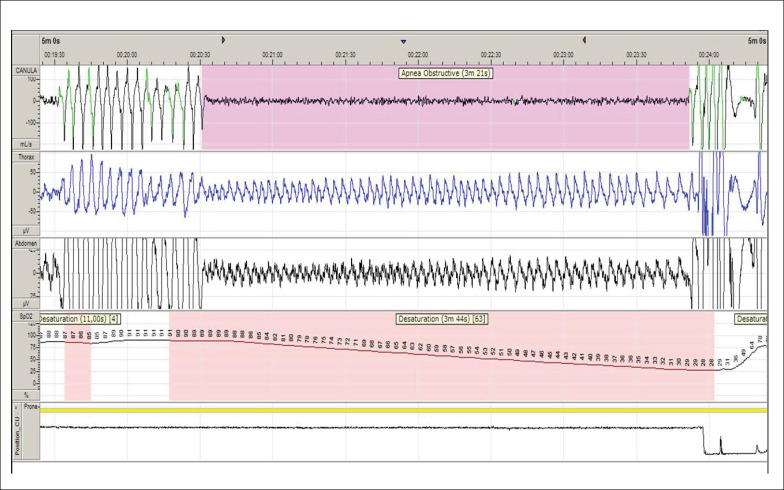
A apneia obstrutiva mais longa durante o monitoramento do sono. Observe a hipoxemia acentuada relacionada e o canal de posição do sensor revelando a posição prona (traço amarelo).

Uma mulher de 63 anos foi internada eletivamente para realizar um procedimento de intervenção coronária percutânea (ICP). Embora ela não se queixasse de sonolência diurna (Epworth Sleepiness Scale: 9), seus parentes a descreveram como muito sonolenta, apresentava roncos altos e pausas noturnas na respiração durante o sono. À inspeção, não havia importantes sinais de alterações crânio-faciais que predispusessem à AOS. O índice de massa corpórea (IMC) estava dentro da normalidade (25 kg/m^2^), mas a pressão arterial (PA) não estava controlada (152/84 mmHg). Curiosamente, uma recente monitorização ambulatorial da PA (MAPA) mostrou um padrão reverso da queda da PA noturna (PA durante o sono igual ou superior à vigília) (Figura 1). Ela estava em uso regular de aspirina, carvedilol, anlodipino e atorvastatina. Os medicamentos para PA foram ajustados pela equipe médica. A paciente foi submetida a uma ICP bem-sucedida na artéria descendente anterior esquerda usando um *stent* convencional. Após o procedimento, ela foi submetida a uma avaliação do sono usando um monitor portátil (Embletta Gold^®^). A paciente apresentava um índice de apneia-hipopneia de 26,7 eventos/hora com a menor saturação periférica de oxigênio (SpO_2_) registrada de 28% e tempo total com SpO_2_ <90% de 33%. Além disso, apresentou apneias muito longas (19 episódios com duração >1 minuto e o evento mais longo com incríveis 3,21 minutos de duração) (Figura 2). Nota-se que a paciente permaneceu 76% do tempo na posição prona (sua posição preferida para dormir). A paciente optou por não procurar nossa clínica ambulatorial do sono, apesar de recomendarmos ativamente o tratamento para a AOS. Após 11 meses da ICP, a paciente sofreu um infarto agudo do miocárdio. Aproximadamente 2 anos após o procedimento de ICP, ela sofreu um episódio de acidente vascular cerebral e quatro meses depois um novo infarto fatal do miocárdio durante um cochilo à tarde (por volta das 15h), apesar de tratamento medicamentoso para a DAC.

## Discussão

Este caso chamou nossa atenção devido à apresentação incomum de AOS em paciente de alto risco cardiovascular: o IMC dentro dos valores de normalidade e os eventos respiratórios muito longos predominantemente na posição prona. Em um estudo anterior, foram relatadas apneias obstrutivas com duração de até 3,89 minutos em um paciente com disfunção autonômica, provavelmente refletindo a falta de controle autonômico protetor na interrupção dos eventos apneicos.^3^ Nossa paciente não apresentava evidência de doença autonômica, apesar da história de diabetes. De fato, não foram observados períodos de hipotensão na MAPA. Em contraste, observamos um padrão noturno reverso da PA na MAPA. Demonstrou-se que o padrão de descenso reverso da PA sistólica. está associada a um aumento de 4 vezes na probabilidade de AOS, independentemente da presença de queixas de sono ou questionários positivos de sono.^4^

Um achado interessante é a ocorrência incomum de eventos obstrutivos na posição prona. É amplamente aceito que a posição supina predispõe a obstruções das vias aéreas superiores durante o sono.^5^ Estudos preliminares relataram a posição prona como terapia adjuvante para AOS.^6^^,^^7^ Por outro lado, este relato de caso destaca que a posição prona pode não ser um espectador inocente, como observado em bebês.^8^ Embora nenhuma relação causa-efeito possa ser comprovada, é concebível que a posição prona combinada com um alto limiar de excitação possa contribuir para eventos muito longos observados durante o sono nesta paciente.

Por fim, vale ressaltar que os pacientes com AOS e DAC pré-estabelecidas não tiveram benefícios da pressão positiva contínua nas vias aéreas (CPAP) na prevenção de doenças cardiovasculares, segundo o estudo SAVE.^9^ A carga acentuada de hipóxia (como observado neste caso) não foi incluída no perfil usual do estudo SAVE, impedindo qualquer conclusão definitiva sobre os benefícios do tratamento da AOS entre pacientes com AOS e importante hipoxemia. A falta de tratamento específico para a AOS neste caso pode ter contribuído para os resultados cardiovasculares observados.

## Conclusão

Este é um caso incomum de apneias muito longas durante a posição prona em uma paciente com o IMC dentro da normalidade e DAC. Conforme sugerido em um estudo observacional multicêntrico, o seguimento desfavorável sugere que a AOS não é um espectador inocente na DAC, principalmente na presença de diabetes.^10^^,^^11^ Portanto, os resultados neutros do estudo SAVE não devem impedir o tratamento visando potenciais benefícios cardiovasculares em pacientes de alto risco com hipoxemia grave.
