# Supplementary material for: Apneias Muito Longas em Posição Prona em uma Paciente Eutrófica com Doença Arterial Coronariana: Implicações para o Risco Cardiovascular
Source: Arq Bras Cardiol. 2021 Feb 2;116(2 Suppl 1):25–7. [Article in Portuguese] doi: 10.36660/abc.20200191 (PMC8118627; doi:10.36660/abc.20200191)
Supplement: Translation

# Translation: Very Long Apneas during Prone Position in a Lean Patient with Coronary Artery Disease: Implications for the Cardiovascular Risk

**Authors:** Sofia Fontanello Furlan, Viktor Sinkunas, Pedro Rodrigues Genta, Geraldo Lorenzi, Luciano F. Drager

**Affiliations:** 1 São PauloSP Brazil Universidade de São Paulo Instituto do Coração, São Paulo, SP – Brazil

**Keywords:** Angioplasty, Sleep Apnea Obstructive, Coronary Artery Disease, Percutaneous Coronary Intervention, Body Mass Index, Obesity, Blood Pressure Monitoring Ambulatory, Oxymetria

## Introduction

Obstructive sleep apnea (OSA) is a common condition characterized by recurrent upper airway obstructions during sleep, promoting intermittent hypoxia and sleep fragmentation.^1^ Traditional risk factors for OSA include male gender and obesity. Overall, patients with severe OSA presented longer events and more severe hypoxemia. Supine sleep is consistently associated with more severe OSA rates in adults.^2^ However, the relationship between OSA indices and prone position is inconsistent.^2^ Here, we reported a very peculiar presentation of OSA characterized by very long respiratory events in a prone position in a lean female patient with a history of high blood pressure, diabetes mellitus, chronic kidney disease under dialysis therapy and a recent diagnosis of coronary artery disease (CAD).

## Case Report

A 63 year-old female was electively admitted to the Hospital to perform a percutaneous coronary intervention (PCI) procedure. Eventhough she did not complain of daytime sleepiness (Epworth Sleepiness Scale: 9), her relatives described she was very sleepy, presented loud snoring and nocturnal breathing pauses during sleep. Upon inspection, there were no important signs of craniofacial changes that predisposed to OSA. Body-mass index (BMI) was within the normal range (25 kg/m^2^) but arterial blood pressure was uncontrolled (152/84 mmHg). Interestingly, a recent ambulatory blood pressure monitoring (ABPM) showed a reverse systolic dipping pattern (blood pressure during sleep equal or higher than wakefulness) (Figure 1). She was under regular use of aspirin, carvedilol, amlodipine and atorvastatin. Arterial blood pressure medications were further adjusted by the medical team. The patient underwent a successful PCI in the *left anterior descending artery* using a bare metal stent. After the procedure, she underwent a sleep study using a portable monitor (Embletta Gold^®^). The patient had an apnea-hypopnea index of 26.7 events/hour with a lowest peripheral oxygen saturation (SpO_2_) of 28% and a total time with a SpO_2_ <90% of 33%. Very long apneas (19 episodes lasting >1 minute and the longest event lasting the incredible 3.21 minutes) (Figure 2) were observed. Of note, the patient spent 76% of the time in the prone position (her preferred sleep position).

**Figure 1:** Ambulatory blood pressure monitoring showing reverse systolic blood pressure dipping pattern. Mean daytime blood pressure: 150x81 mmHg; Mean nighttime blood pressure: 155x79 mmHg.

**Figure 2:** The longest obstructive apnea during sleep monitoring. Observe the severe related hypoxemia and the sensor position channel revealing prone position (yellow trace)

The patient did not seek our outpatient sleep clinic despite we actively recommended treatment for OSA. After 11 months of the PCI, the patient suffered an acute myocardial infarction. Approximately 2 years after the PCI procedure, she suffered an episode of stroke and four months later a new fatal myocardial infarction during a nap at 3:00 pm despite using standard medications for CAD.

## Discussion

This case called our attention due to the unusual presentation of OSA in a high cardiovascular risk patient: BMI in the normal range and very long respiratory events predominantly in the prone position. In a previous study, obstructive apneas lasting up to 3.89 min in a patient with autonomic dysfunction was reported, probably reflecting the lack of protective autonomic control in terminating the apneic events.^3^ Our patient had no evidence of autonomic disease, despite the history of diabetes. Indeed, no periods of hypotension were observed in the ABPM. In contrast, we observed a reverse dipping pattern in the ABPM. Reverse dipping has been shown to be associated with a 4-fold increase in the probability of significant OSA, regardless the presence of sleep complaints or positive sleep questionnaires.^4^

One interesting finding is the unusual occurrence of obstructive events in the prone position. It is largely accepted that supine position predisposes to upper airway obstructions during sleep.^5^ Preliminary studies reported prone position as adjunctive therapy for OSA.^6^^,^^7^ In contrast, this case report underscore that prone position may not be an innocent bystander, as observed in infants.^8^ Although no cause-effect relationship may be proven, it is conceivable that the prone position combined with a high arousal threshold may contribute to very long events observed during sleep in this patient.

Finally, it is worthy to mention that OSA patients with CAD had no benefits of continuous positive airway pressure (CPAP) in preventing cardiovascular disease according to the SAVE trial.^9^ Severe hypoxia burden (as observed in this case) was not included in the usual SAVE study's profile, preventing any definitive conclusion on the benefits of OSA treatment among OSA patients with severe hypoxia burden. The lack of specific OSA treatment may have contributed to the observed cardiovascular outcomes.

## Conclusions

This is an unusual case of very long apneas during prone position in a lean patient with CAD. As suggested in a multicenter observational study, the unfavorable follow-up suggests that OSA is not an innocent bystander in CAD, especially in the presence of diabetes.^10^^,^^11^ Therefore, the neutral results of SAVE trial should not preclude treatment aiming potential cardiovascular benefits in high risk patients with severe hypoxemia.
